# MOVIE phase II trial of tremelimumab plus durvalumab combined with metronomic oral vinorelbine in patients with head and neck cancer

**DOI:** 10.1016/j.esmoop.2025.105840

**Published:** 2025-11-06

**Authors:** E. Borcoman, A. Hervieu, C. Cropet, E. Coquan, J. Guigay, F. Rolland, M. Bernadach, E. Charafe, F. Legrand, E. Dassé, C. Le Tourneau, A. Gonçalves

**Affiliations:** 1 Department of Drug Development and Innovation (D3i), Institut Curie, Paris-Saclay University, Paris, France; 2 Deparment of Medical Oncology, Centre George François Leclerc, Dijon, France; 3 Statistical Unit, Clinical Research Department, Centre Léon Bérard, Lyon, France; 4 Early Phase Trials Unit, Centre François Baclesse, Caen, France; 5 Centre Antoine Lacassagne, Nice, France; 6 Deparment of Medical Oncology, Institut Cancérologie de l’Ouest, Nantes, France; 7 Deparment of Medical Oncology, Centre Jean Perrin, Clermont Ferrand, France; 8 Deparment of Pathology, Institut Paoli-Calmettes, Marseille, France; 9 Unicancer, Paris, France; 10 Aix-Marseille Univ, Centre de Recherche en Cancérologie de Marseille (CRCM), INSERM U1068, CNRS U7258, Department of Medical Oncology, Institut Paoli-Calmettes, Marseille, France

**Keywords:** head and neck squamous cell carcinoma (HNSCC), anti-PD-1/PD-L1, anti-CTLA-4, immune checkpoint inhibitor (ICI), metronomic chemotherapy (MC)

## Abstract

**Background:**

Treatment options for advanced solid tumors are limited. In recent years, anti-programmed cell death protein 1/programmed death-ligand 1 (PD-1/PD-L1) immunotherapy as monotherapy has shown significant efficacy, albeit in a limited subset of patients. In the MOVIE trial, metronomic chemotherapy was combined with two immune checkpoint inhibitors (ICIs) to improve clinical outcomes in patients with advanced solid tumors.

**Patients and methods:**

MOVIE was a phase I/II French, multicenter, open-label, nonrandomized study with a Bayesian design that evaluated the antitumor activity and safety of metronomic vinorelbine associated with durvalumab plus tremelimumab. Here, we report on the cohort of patients with head and neck squamous cell carcinoma (HNSCC) from the MOVIE phase II study. Patients were aged ≥18 years with histologically confirmed recurrent or metastatic HNSCC, resistant to conventional therapies, and presented a measurable disease according to RECIST version 1.1. They received oral vinorelbine 40 mg three times a week, and durvalumab 1500 mg plus tremelimumab 75 mg via intravenous infusion on day 1 of 28-day cycles, for a maximum of four cycles of tremelimumab. The primary endpoint was the clinical benefit rate (CBR), defined as the rate of complete response (CR), partial response (PR), or stable disease (SD) lasting at least 24 weeks.

**Results:**

Fifteen HNSCC patients were included between May 2019 and October 2020. The mean estimated CBR according to a noninformative prior distribution was 23.5% (95% credible interval 7.3-45.6). One patient achieved CR, 1 PR, and 1 SD > 24 weeks, leading to an objective response rate of 14.3%. The median progression-free survival was 1.8 months (95% confidence interval 1.0-1.9 months), and the median overall survival was 8 months (95% confidence interval 2.5-12.7 months). The most common vinorelbine-related grade ≥3 adverse events were anemia (*n* = 2, 13%) and neutropenia (*n* = 3, 20%). The most common ICI-related grade ≥3 adverse events were anemia (*n* = 1) and hypokalemia (*n* = 1). There were no treatment interruptions for toxicity and no treatment-related deaths.

**Conclusions:**

Metronomic vinorelbine in combination with dual durvalumab plus tremelimumab immunotherapy had only moderate activity in pretreated advanced HNSCC.

## Highlights

• Metronomic oral vinorelbine combined with durvalumab and tremelimumab was evaluated in refractory HNSCC patients.
• Efficacy was limited with an objective response rate of 14% and a median overall survival of 8 months.
• The toxicity profile was manageable, with the main grade 3+ adverse events being hematological.

## Introduction

Head and neck squamous cell carcinoma (HNSCC) regroups a heterogeneous range of tumors arising from multiple primary sites. HNSCC accounts for 90% of the head and neck cancers, and is the seventh most common cancer worldwide, according to the 2020 GLOBOCAN estimates, with an estimated 890 000 new cases and 450 000 deaths per year.^1^, ^2^, ^3^ Its global incidence continues to rise with a predicted 30% annual increase by 2030.^2^ This incidence varies across countries and has been predominantly correlated with tobacco exposure, excessive alcohol intake, and infection with human papillomavirus.^4^ Most patients have locally advanced tumors at diagnosis with a high risk of recurrence.^5^ Therapeutic approaches usually include a combination of surgery, radiotherapy, chemotherapy, and molecular targeted therapy.^6^^,^^7^ More recently, the introduction of immunotherapy has changed the management of HNSCC with the approval of anti-programmed cell death protein 1 (PD-1) immune checkpoint inhibitors (ICIs) pembrolizumab and nivolumab for the treatment of patients with recurrent or metastatic HNSCC in first line and in platinum-refractory disease.^6^^,^^7^

As shown in advanced melanoma or renal cell carcinoma,^8^^,^^9^ the efficacy of immunotherapy may be enhanced when different strategies are combined and various targets are modulated.^10^ Among these approaches, dual immune checkpoint blockage targeting the cytotoxic T lymphocyte-associated protein 4 (CTLA-4) and PD-1 is currently being tested. Indeed, the mechanisms of CTLA-4 and PD-1 pathways are not redundant, suggesting that targeting of both pathways may have additive or synergistic activity. Recently, the association of anti-PD-1 with anti-CTLA-4 has demonstrated a major pathological response of 35% when given before surgery in patients with locoregionally advanced HNSCC.^11^

The promising results obtained with ICIs have led to the emergence of trials that explore treatment strategies incorporating immunotherapy and chemotherapy.^12^^,^^13^ In HNSCC, multiple studies have reported high response rates.^14^, ^15^, ^16^ However, the combination of immunotherapy, composed of several ICIs, and chemotherapy is clearly associated with a higher risk of adverse reactions.

Because of its excellent tolerance and favorable toxicity profile, metronomic chemotherapy (MC) is an emerging therapeutic alternative in clinical oncology. MC relies on the administration of low doses of cytotoxic agents over an extended period.^17^, ^18^, ^19^ Interestingly, although the metronomic administration of chemotherapy is supposed to primarily target tumor angiogenesis, immunomodulation also occurs, shifting the immunological balance from immunosuppression to immunostimulation through various mechanisms.^20^ Several chemotherapeutic agents have been evaluated using metronomic schedules in solid tumors, including vinorelbine, a vinca alkaloid that inhibits microtubule polymerization during mitosis, thereby preventing cell proliferation.^20^, ^21^, ^22^ Furthermore, vinorelbine has been reported to have immunomodulatory properties at low doses.^22^

MOVIE was a phase I/II basket trial which evaluated a combination of metronomic oral vinorelbine plus anti-programmed death-ligand 1 (PD-L1)/anti-CTLA-4 immunotherapy in patients with advanced solid tumors. The phase I part of the study established the feasibility of combining metronomic vinorelbine with durvalumab and tremelimumab. In view of the preliminary efficacy results (half of the patients included at the recommended dose for phase II achieved disease control) and reassuring toxicity profile, the study was moved toward phase II.^23^ Here, we present the results of the phase II part of the MOVIE trial in which the antitumor activity and safety of oral metronomic vinorelbine combined with dual anti-PD-L1 durvalumab and anti-CTLA-4 tremelimumab immunotherapy were assessed in pretreated advanced HNSCC patients.

## Methods

### Study design and participants

The MOVIE trial was a phase I/II national, multiple cohort, multicenter, open-label, nonrandomized trial using a Bayesian design that aimed to evaluate activity and safety of metronomic oral vinorelbine with durvalumab plus tremelimumab in patients with advanced solid tumors including head and neck, prostate, cervix, breast cancers, and miscellaneous malignancies with high mutational load and/or microsatellite instability-high. This study was divided into two successive parts: a phase I dose escalation expanded to a phase II to assess the activity and safety of the vinorelbine and durvalumab plus tremelimumab combination under the previously defined recommended phase II dose (RP2D). MOVIE phase I was conducted from 18 July 2018 to 15 May 2019.^23^ Patients treated at RP2D during phase I were considered assessable for phase II. MOVIE phase II was conducted from 6 June 2019 to 14 September 2021.

Patients included in the head and neck cohort were men and women aged ≥18 years with histologically confirmed recurrent or metastatic HNSCC, resistant to conventional therapies, and candidates for experimental therapy according to the local clinical board. Eligible patients were required to have a measurable disease by RECIST version 1.1, an Eastern Cooperative Oncology Group (ECOG) performance status (PS) ≤1, normal hematological (absolute neutrophil count ≥1.5 × 10^9^/l; platelets count ≥100 × 10^9^/l; hemoglobin ≥9.0 g/dl), hepatic [total bilirubin ≤1.5 upper limit of normal (ULN) unless documented Gilbert’s syndrome; aspartate aminotransferase and alanine aminotransferase ≤2.5 ULN/≤5 ULN in the presence of liver metastases], and cardiac functions (left ventricular ejection fraction ≥50%), measured creatinine clearance (Cockcroft and Gault) ≥40 ml/min or creatinine ≤1.5 times ULN, evidence of postmenopausal status or negative urinary or serum pregnancy test for female premenopausal patients, and an estimated life expectancy of at least 3 months. Exclusion criteria were other concurrent malignancies, active brain metastases, spinal cord compression, or leptomeningeal disease (except local meningeal disease due to local recurrence), previous treatment with an anti-PD-1 or anti-PD-L1 antibody or vinorelbine (in advanced settings), current or prior use of immunosuppressive medication within 14 days before the first dose of durvalumab or tremelimumab, prior anticancer therapy within the past 3 weeks, major surgery within 28 days before the first dose of study treatment, and participation in another clinical trial with an investigational product within 21 days of inclusion.

### Treatment and assessments

RP2D was defined during phase I of the MOVIE study with oral vinorelbine 40 mg three times a week, and durvalumab 1500 mg plus tremelimumab 75 mg via intravenous infusion on day 1 of 28-day cycles (every 4 weeks).^23^ Patients received vinorelbine until disease progression, and up to 26 cycles of durvalumab and four cycles of tremelimumab. Dose reductions of durvalumab and tremelimumab were not permitted. Patients were treated until progression of their disease, unacceptable toxicity, intercurrent conditions that precluded continuation of treatment, or their refusal. During durvalumab plus tremelimumab combination, dose interruptions were always applied to both compounds. Clinical disease and radiologic assessments were carried out at baseline, at day 28 every two cycles during the treatment phase, then at the end of treatment visit, and every 12 weeks for up to 2 years thereafter. Radiological response was assessed by investigators, including local radiologists, according to RECIST version 1.1. Tumor evaluation had to be continued during the post-treatment period if withdrawal was not related to disease progression and should have been continued and documented every 8 weeks (or every 12 weeks after the first 12 months of the treatment phase) until disease progression or initiation of an antineoplastic treatment. Information concerning adverse events (AEs) was recorded from the time of signature of informed consent, throughout the treatment period, and including the follow-up period (30 days after the last administration of vinorelbine or for 90 days after the last administration of durvalumab and tremelimumab, whichever period was longer). AEs were graded according to the National Cancer Institute-Common Terminology Criteria for Adverse Events (NCI-CTCAE) version 5.0.

### Outcomes

The primary endpoint of phase II of the MOVIE study was the clinical benefit rate (CBR) defined as the rate of complete response (CR), partial response (PR), or stable disease (SD) lasting at least 24 weeks according to RECIST version 1.1. Secondary objectives were safety, objective response rate (ORR), duration of response (DOR), progression-free survival (PFS), and overall survival (OS). The assessment of safety rested on the frequency and severity of AEs based on the common toxicity criteria grade (NCI-CTCAE version 5.0). ORR was defined as the percentage of CR or PR as the best response measured according to RECIST version 1.1, duration of response was measured as the period from documented tumor response (CR/PR) to disease progression by RECIST version 1.1 or death from any cause. PFS was measured from the first administration of the combination immunotherapy to the first documented progression by RECIST or death from any cause. Patients alive who had not progressed at the time of the analysis were censored at the time of their last available assessment. OS was measured from the first administration of the combination immunotherapy to death from any cause. Patients alive at the time of the analysis were censored at the time of their latest visit.

### Evaluation of PD-L1 expression

Formalin-fixed paraffin-embedded tumor specimens were collected to monitor PD-L1 expression by immunohistochemistry using the IHC 22C3 pharmDx assay (Agilent Technologies). The evaluation of PD-L1 expression was carried out by independent pathologists, who were blinded to the clinicopathological data, including the therapeutic response and survival time. PD-L1 positivity or overexpression was defined as a combined positive score (CPS) ≥1.

### Statistical analysis

The analysis of the primary endpoint (CBR) was carried out sequentially using a Bayesian approach with a beta-binomial model. A maximum sample size of 30 patients was included. Interim analyses were planned after a 24-week follow-up of the first 10 patients of each cohort and then every 5 patients. The Bayesian approach was based on an update, at each interim analysis, of our baseline knowledge of the CBR, which was before the beginning of the study. Three ‘prior’ probability models (distributions) for the CBR were defined in the protocol (1, a noninformative prior; 2, an informative optimistic prior centered on a CBR of 30%; and 3, a less informative optimistic prior centered on a CBR of 30%). A stopping criterion for inefficacy was also defined, and stopping the HNSCC cohort could have been recommended during interim analyses if there was a high probability (≥0.75) that the estimated CBR was less than or equal to the futility bound (p0 = 20%). The first interim analysis of the HNSCC cohort was conducted in May 2021 on 13 assessable patients and did not meet the futility criterion. However, the recruitment of the cohort was stopped following the publication of the KESTREL study data.^24^ Here, we present the final analysis carried out on the 15 HNSCC assessable patients enrolled in the MOVIE study at the time the cohort was stopped.

ORR was presented with the associated 95% confidence intervals (CIs). PFS, OS, and duration of response were estimated using the Kaplan–Meier method and described in terms of medians with the associated 95% CI. The analysis was carried out on the full analysis set (FAS) population consisting of all included patients having received at least one cycle of study treatment or discontinued the treatment before the end of the first cycle for progression or toxicity (treatment failures), with no major protocol violation that could have biased primary endpoint evaluation. The safety analysis was conducted on all patients who had received at least one dose of the study drugs.

### Ethics approval and consent to participate

The study was sponsored by Unicancer and conducted in the rigorous standards set out in the Good Clinical Practice guidelines and in accordance with the principles in the Declaration of Helsinki. The study protocol was approved by the French Ethical Committee CPP Sud-Méditerranée I and by the French regulatory authorities. Patients had to confirm their consent in writing before starting the study and before any study-related procedures. It is registered at ClinicalTrials.gov (identifier: NCT03518606).

## Results

### Patients and treatment

Between May 2019 and October 2020, 15 patients from France were enrolled in the HNSCC cohort of the MOVIE study. All patients were included in the FAS and safety populations. Data cut-off date for the analysis was 21 July 2023. Patients and disease characteristics, and type of prior therapies are described in Table 1. The median age was 58 years (range 41-70 years). Out of 15 patients, 13 (87%) were men and 2 (13%) were women. ECOG at baseline was 0 for 5 (33%) patients and 1 for 10 (67%) patients. The primary site of cancer was the oropharynx for 6 (40%) patients, the oral cavity for 4 (27%), the hypopharynx for 2 (13%), and the larynx for 3 (20%). Eight (53%) patients had distant metastases at inclusion, and the disease was locally advanced for 7 (47%) patients. Metastatic sites at inclusion were mainly located in lymph nodes (*n* = 9, 60%) and lung (*n* = 5, 33%). All patients had previously received chemotherapy, most had radiotherapy (*n* = 11, 73%), and surgery (*n* = 10, 67%). At the data cut-off date, all patients had either definitely discontinued treatment due to progression (*n* = 12, 80%), death (*n* = 2, 13%), or a physician’s decision (*n* = 1, 7%). The median duration of exposure to vinorelbine and immunotherapy was 2.8 and 3 months, respectively.

**Table 1:** Patient and disease characteristics

Characteristics	Cohort
	Head and neck cancer (*N* = 15)
Age (years; at inclusion)	
Number of patients	15
Mean (standard deviation)	56.7 (8.1)
Median (range)	58 (41-70)
Sex, *n* (%)	
Male	13 (87)
Female	2 (13)
Eastern Cooperative Oncology Group (ECOG) at baseline, *n* (%)	
0	5 (33)
1	10 (67)
Time from initial diagnosis to inclusion (years)	
Number of patients	15
Missing, *n*	0
Mean (standard deviation)	1.9 (1.5)
Median (range)	1.4 (0.3-5.5)
Disease status at inclusion, *n* (%)	
Metastatic with or without locoregional recurrence	8 (53)
Locally advanced^a^	7 (47)
Classification M at diagnosis, *n* (%)	
M0	14 (93)
M1	1 (7)
p16 status at diagnosis, *n* (%)	
Missing, *n*	4
p16 positive	5 (46)
p16 negative	6 (55)
Primary site of cancer, *n* (%)	
Oral cavity	4 (27)
Hypopharynx	2 (13)
Oropharynx	6 (40)
Larynx	3 (20)
Metastatic sites at screening, *n* (%)	
Liver	1 (7)
Bone	3 (20)
Pleura	2 (13)
Lung	5 (33)
Lymph nodes^b^	9 (60)
Retroperitoneum	1 (7)
Locoregional recurrence^c^	4 (27)
Number of previous metastatic lines, *n* (%)	
0	7 (46.7)
1	7 (46.7)
2	1 (6.7)
Previous treatment received, *n* (%)	
Chemotherapy	15 (100)
5-FU	10 (67)
Carboplatin	7 (47)
Cisplatin	14 (93)
Docetaxel	6 (40)
Other	5 (33)
Radiotherapy	11 (73)
Local radiotherapy	10 (67)
Radiotherapy at metastatic sites	4 (27)
Surgery	10 (67)
Tumorectomy	5 (33)
Other	7 (47)
Targeted therapy	6 (40)
Cetuximab	6 (40)
Reason for treatment discontinuation, *n* (%)	15 (100)
Progressive disease	12 (80)
Physician decision	1 (7)
Death	2 (13)

a Locally advanced refers to cancer that has spread from where it started to nearby tissue or lymph nodes.

b Lymph nodes as metastatic sites refer to lymph nodes that contain cancer, which has spread from somewhere else in the body.

c Locoregional recurrence means in the cervical area at the T or N levels.

### Efficacy

**Figure 1:**
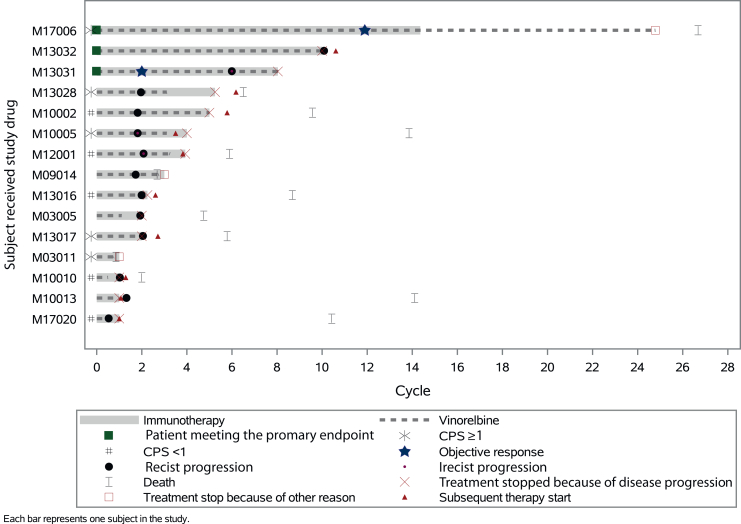
**Swimmer plot displaying efficacy results.** Each bar represents one patient from the time of their inclusion to the data cut-off date (21 July 2023). In total, 15 patients were included in the head and neck squamous cell carcinoma cohort of the MOVIE trial and received vinorelbine 40 mg three times a week, durvalumab 1500 mg Q4W, and tremelimumab 75 mg Q4W. CPS, combined positive score; Q4W, every 4 weeks.

A swimmer plot (Figure 1) summarizes each patient’s response to treatment over the course of the study. Among the FAS population, 3/15 (20%) patients experienced a clinical benefit (Table 2). The mean estimated CBR (95% credible interval) according to the prior distribution (i.e. 1, noninformative prior; 2, informative optimistic prior; and 3, less informative optimistic prior) was 23.5% (7.3-45.6), 22.9% (8.0-42.5), and 21.4% (6.2-42.8), respectively. There was 1 CR, 1 PR, 1 SD ≥24 weeks, 11 (78.6%) PD, and 1 patient was not assessable (died from septic shock before any tumoral evaluation) as the best response. ORR was estimated at 14.3% and the duration of response for the CR and PR patients was 13.6 and 3.7 months, respectively. In these patients, the time to first observed objective response was 10.9 and 1.8 months. PFS events were reported for all the patients with 13 disease progressions, one death due to progressive disease, and one death from septic shock (Supplementary Figure S1, available at https://doi.org/10.1016/j.esmoop.2025.105840). The median PFS was 1.8 months (95% CI 1.0-1.9 months). At data cut-off, 13 patients (87%) had died due to disease progression (*n* = 11; 73%), intercurrent disease (*n* = 1; 7%), and septic shock (*n* = 1; 7%). The median follow-up for OS and median OS were 8.3 months (95% CI 7.4-9.3 months) and 8.0 months (95% CI 2.5-12.7 months), respectively (Supplementary Figure S2, available at https://doi.org/10.1016/j.esmoop.2025.105840).

**Table 2:** Summary of efficacy endpoints

Summary	Head and neck squamous cell carcinoma cohort (*N* = 15)
**CBR** [Bayesian estimation/mean estimated success rate (95% credible interval)]	
Success^a^ (CR + PR + SD ≥24 weeks), *n*	3/15
Prior distribution 1, % Prior noninformative distribution = beta (1-1)	23.5 (7.3-45.6)
Prior distribution 2, % Prior informative optimistic distribution = beta (1.8-4.2)	22.9 (8.0-42.5)
Prior distribution 3, % Prior less informative optimistic distribution = beta (0.75-1.75)	21.4 (6.2-42.8)
**Best overall response, *n* (%)**	
CR	1 (7.1)
PR	1 (7.1)
SD ≥24 weeks	1 (7.1)
PD	11 (78.6)
Not assessable	1^b^
Objective response rate (CR + PR)	2 (14.3)

CBR, clinical benefit rate; CR, complete response; PD, progressive disease; PR, partial response; SD, stable disease.

a The CBR was estimated based on a binary variable (success/failure) defined as follows: Success, patients with CR or PR as best response status obtained until 24 weeks after inclusion; patients with SD as best response status obtained until 24 weeks after starting treatment: success, if no RECIST progression, nor definite treatment discontinuation for progression or death at the time of the analysis, and the duration between inclusion and the last tumoral evaluation was ≥22 weeks; success, if a RECIST progression occurred >26 weeks after inclusion; failure, if a RECIST progression occurred within 26 weeks after inclusion; failure, if treatment was discontinued for progression or death within 26 weeks after inclusion, and no tumoral evaluation was carried out after treatment discontinuation; failure, patients with PD as best response status obtained until 24 weeks after inclusion; failure, patients having definitely discontinued treatment for progression or death before any tumoral evaluation.

b One patient died from septic shock before any tumoral evaluation.

### PD-L1 assessment

PD-L1 expression was assessed in available tumor tissue of 10 patients. In total, 5 (50%) were determined to have positive PD-L1 (CPS ≥1) expression (none had CPS ≥10). The patient who experienced a CR was PD-L1 (CPS ≥1), whereas the one with a PR was not assessable for PD-L1 expression.

### Safety

Table 3 provides a summary of all AEs, and Table 4 describes vinorelbine-related and ICI-related grade ≥2 AEs. All patients reported at least one grade ≥2 AE. In total, 5 (33%) presented at least one grade ≥3 treatment-related AE (TRAE) and 12 (80%) one serious AE. There were two G5 AEs reported that were not related to any study treatment but probably linked to the disease progression (1 septic shock and 1 tumor hemorrhage). The most common vinorelbine-related grade ≥3 AEs were anemia (*n* = 2, 13%) and neutropenia (*n* = 3, 20%). The most common ICI-related grade ≥3 AEs were anemia (*n* = 1) and hypokalemia (*n* = 1). A total of 7 (47%) patients experienced at least one interruption of vinorelbine, 1 patient had one dose reduction of vinorelbine, and 2 (13%) patients had one dose delay due to toxicity. Tremelimumab was delayed due to toxicity in 2 (13%) patients and durvalumab in 3 (20%) patients. No TRAEs led to treatment discontinuation.

**Table 3:** Summary of AEs

Summary	Head and neck squamous cell carcinoma (*N* = 30), *n* (%)
AE	15 (100.0)
Vinorelbine-related AE	11 (73.3)
ICI-related AE	11 (73.3)
Treatment-related AE	12 (80.0)
Grade ≥2 AE	15 (100.0)
Grade ≥2 vinorelbine-related AE	10 (66.7)
Grade ≥2 ICI-related AE	8 (53.3)
Grade ≥2 treatment-related AE	11 (73.3)
Grade ≥3 AE^a^	13 (86.7)
Grade ≥3 vinorelbine-related AE	5 (33.3)
Grade ≥3 ICI-related AE	2 (13.3)
Grade ≥3 treatment-related AE	5 (33.3)
Grade ≥5 AE	2 (13.3)
Grade ≥5 vinorelbine-related AE	0 (0)
Grade ≥5 ICI-related AE	0 (0)
Grade ≥5 treatment-related AE	0 (0)
SAE	12 (80.0)
Vinorelbine-related SAE	3 (20)
ICI-related SAE	0 (0)
Treatment-related SAE	3 (20)

AE, adverse event; ICI, immune checkpoint inhibitor; SAE, serious adverse event.

a Only 1 grade 4 AE was reported and related to vinorelbine (neutropenia).

**Table 4:** Description of treatment-related grade ≥2 adverse events

Adverse event description	Preferred term	Grade max	Head and neck squamous cell carcinoma (*N* = 15), *n* (%)
Vinorelbine-related grade ≥2 adverse events			
Blood and lymphatic system disorders	Anemia	Total	3 (20.0)
		2	1 (6.7)
		3	2 (13.3)
	Lymphopenia	Total	2 (13.3)
		2	2 (13.3)
	Neutropenia	Total	3 (20.0)
		2	1 (6.7)
		3	2 (13.3)
		4	1 (6.7)
General disorders and administration site conditions	Asthenia	Total	2 (13.3)
		2	2 (13.3)
	Face edema	Total	1 (6.7)
		2	1 (6.7)
	Pain	Total	1 (6.7)
		2	1 (6.7)
Infections and infestations	Sepsis	Total	1 (6.7)
		3	1 (6.7)
Metabolism and nutrition disorders	Cell death	Total	1 (6.7)
		2	1 (6.7)
	Decreased appetite	Total	1 (6.7)
		2	1 (6.7)
	Hypokalemia	Total	1 (6.7)
		3	1 (6.7)
	Hypophosphatemia	Total	1 (6.7)
		2	1 (6.7)
Neoplasms benign, malignant, and unspecified (including cysts and polyps)	Tumor hemorrhage	Total	1 (6.7)
		2	1 (6.7)
Blood and lymphatic system disorders	Anemia	Total	1 (6.7)
		3	1 (6.7)
	Lymphopenia	Total	1 (6.7)
		2	1 (6.7)
General disorders and administration site conditions	Asthenia	Total	2 (13.3)
		2	2 (13.3)
	Face edema	Total	1 (6.7)
		2	1 (6.7)
	Pain	Total	1 (6.7)
		2	1 (6.7)
Investigations	Lipase increased	Total	1 (6.7)
		2	1 (6.7)
	Decreased appetite	Total	1 (6.7)
		2	1 (6.7)
	Hypokalemia	Total	1 (6.7)
		3	1 (6.7)
	Hypophosphatemia	Total	1 (6.7)
		2	1 (6.7)
	Other	Total	1 (6.7)
		2	1 (6.7)
Neoplasms benign, malignant, and unspecified (including cysts and polyps)	Tumor hemorrhage	Total	1 (6.7)
		2	1 (6.7)
Respiratory, thoracic, and mediastinal disorders	Alveolitis allergic	Total	1 (6.7)
		2	1 (6.7)

## Discussion

As therapeutic options are limited for patients with advanced solid tumors resistant to conventional treatments, the investigators of the MOVIE study hypothesized that MC would potentiate the efficacy of dual ICI in patients with recurrent or metastatic HNSCC. During the phase I part of the study, the feasibility of combining oral metronomic vinorelbine at 40 mg three times a week with monthly intravenous durvalumab at 1500 mg and tremelimumab at 75 mg was established.^23^ Among the 14 patients enrolled at the time, preliminary data showed one patient who presented a CR and four patients with SD. In the MOVIE phase II study, five different cohorts, including HNSCC along with four others, were tested. The Bayesian sequential design, including interim analyses, allowed for the termination of each cohort due to futility before trial completion. The first interim analysis of the HNSCC cohort was carried out on 13 assessable patients and did not meet the stopping criterion. However, the cohort was stopped shortly afterward following the results of the KESTREL study (NCT02551159).^24^ Indeed, KESTREL did not demonstrate the superiority of durvalumab or durvalumab plus tremelimumab compared with the standard of care (EXTREME regimen) in terms of OS as first-line treatment in advanced HNSCC patients with tumors expressing a high level of PD-L1. Similarly, the EAGLE (NCT02369874) and Checkmate 651 (NCT02741570) trials, respectively, failed to display longer OS in HNSCC patients treated with durvalumab plus tremelimumab versus standard of care and nivolumab (anti-PD-1) plus ipilimumab (anti-CTLA-4) versus the EXTREME regimen.^25^^,^^26^ The latter study suggested that patients with PD-L1 CPS ≥20 expression may experience a greater effect with immunotherapy; however, the results were not statistically significant in any population. This lack of significance might be partly explained by the number of PD-L1-high patients, which was lower than the number of events required to maintain the planned statistical power for the trial’s primary endpoint. In the current analysis, which included 15 patients with HNSCC, 2 patients who received second-line metronomic vinorelbine associated with durvalumab plus tremelimumab dual immunotherapy achieved CR and PR, while 1 patient had SD >24 weeks, leading to an ORR of 14.3%. Interestingly, the patient who achieved CR had a response lasting >12 months and a PD-L1 CPS ≥1. Taken together, these data suggest that even though most recurrent or metastatic HNSCC patients seemed resistant, ICIs may still be effective in specific HNSCC subpopulations. Whether the presence of biomarkers, such as PD-L1 or tumor mutational burden, could guide the selection is currently being explored.^25^^,^^27^, ^28^, ^29^ So far, immunotherapies targeting PD-(L)1 have demonstrated OS benefits in patients with platinum-refractory or platinum-eligible advanced HNSCC.^30^, ^31^, ^32^

As only a few patients benefit from ICB, research is currently focused on increasing patient responsiveness by developing novel combination strategies to improve their effectiveness. One of the main safety concerns with the use of combination therapies is the increased risk of side-effects.^33^ In the MOVIE study, the association of MC, which has a relatively low toxicity, with dual immunotherapy reduced the likelihood of inducing adverse reactions. Here, we demonstrate that the toxicity of this association was consistent with that expected when using dual immunotherapy, and no new toxicities were identified. If the relatively small sample size does not allow for a conclusion on the safety of the association, it seems that the rate of G3-4 TRAEs was similar to that of the durvalumab plus tremelimumab arm in the KESTREL (19%) or EAGLE trials (16%).^24^^,^^25^ Most importantly, there was no G5 TRAE and no discontinuation of treatment, suggesting that the addition of MC did not amplify the toxicity associated with the use of ICB combination in pretreated advanced HNSCC patients. Originally designed to overcome drug resistance, MC also has the potential for immunomodulation. However, given the moderate activity of the treatment described herein, which was similar to that of a single ICI in pretreated advanced HNSCC, no conclusion can be drawn on its synergistic interaction with immunotherapy. Further investigation will be needed to consolidate the data obtained on such an association, but MC could represent an advantageous treatment option in combination with immunotherapy, especially a dual immunotherapy regimen, in HNSCC patients who have limited therapeutic options.^34^ Furthermore, if there might be an advantage to increasing patient responsiveness to immunotherapy, the timing of immunotherapy relative to other treatments may also affect its efficacy.^35^

In conclusion, although the combination of MC chemotherapy with dual anti-PD-L1/anti-CTLA-4 immunotherapy may offer a valuable treatment option, patients with HNSCC derive moderate benefit from this strategy, comparable to that observed with single ICI in these tumors.
