# Supplementary figures and images for: MOVIE phase II trial of tremelimumab plus durvalumab combined with metronomic oral vinorelbine in patients with head and neck cancer
Source: ESMO Open. 2025 Nov 6;10(11):105840. doi: 10.1016/j.esmoop.2025.105840 (PMC12639439; doi:10.1016/j.esmoop.2025.105840)

**Supplementary Figure 1 : Progression-free survival**


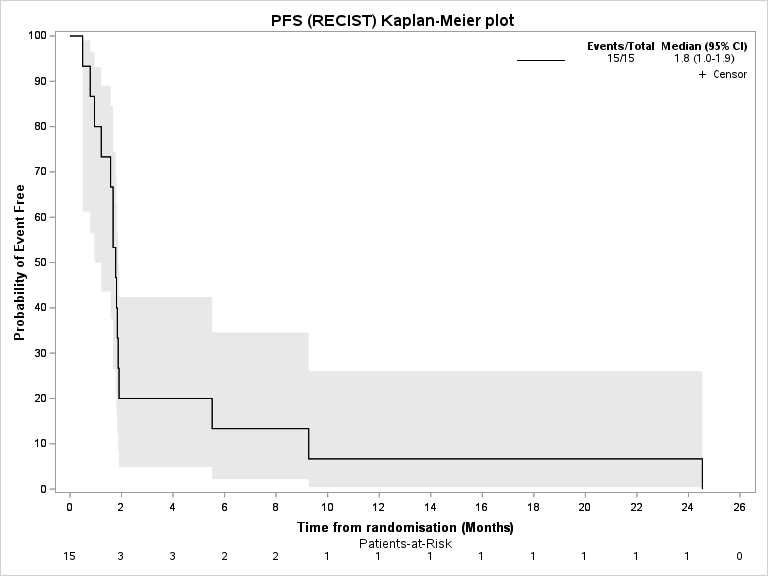


**Supplementary Figure 2 : Overall Survival**


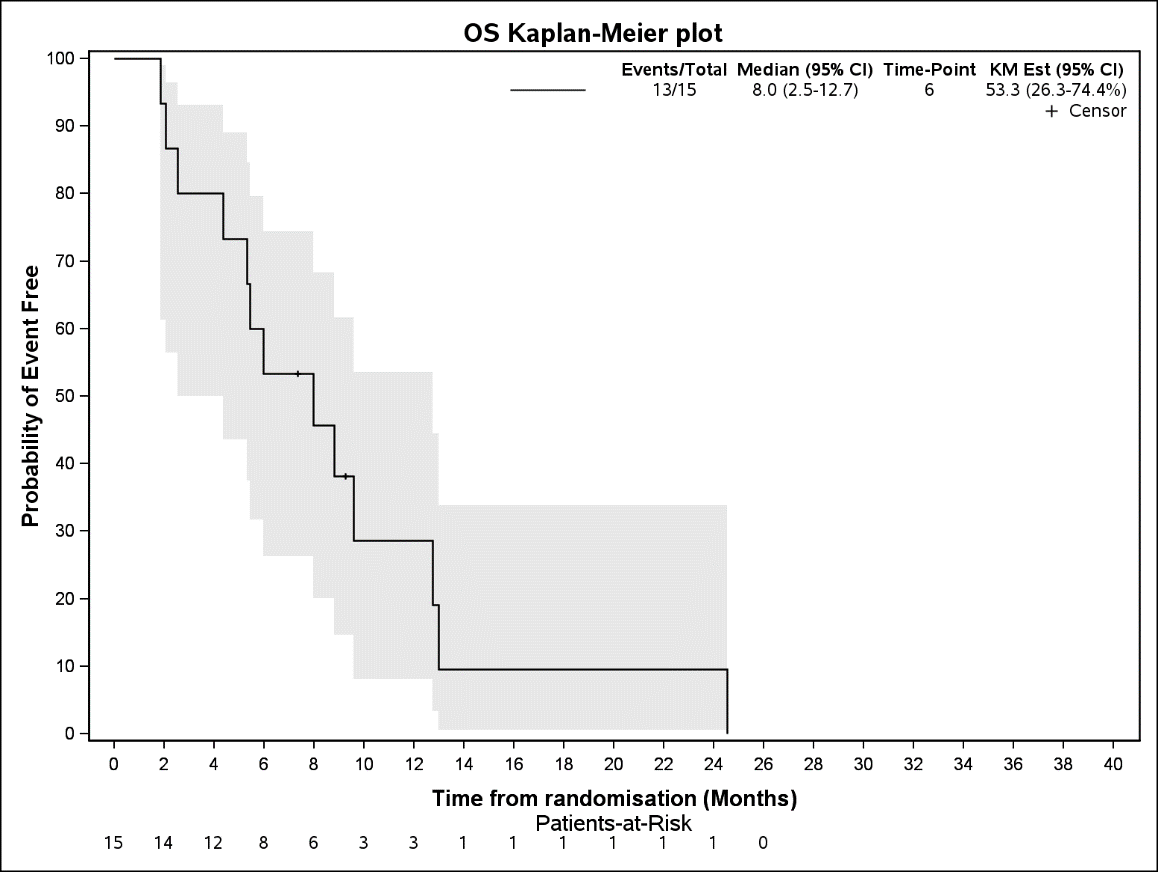

Supplement: Supplementary Figures [file mmc1.docx]
